# Knockout of the *adp* gene related with colonization in *Bacillus nematocida* B16 using customized transcription activator-like effectors nucleases

**DOI:** 10.1111/1751-7915.12282

**Published:** 2015-04-24

**Authors:** Qiuhong Niu, Haoying Zheng, Lin Zhang, Fujun Qin, Loryn Facemire, Guo Zhang, Feng Cao, Ke-qin Zhang, Xiaowei Huang, Jianwei Yang, Lei He, Chanjuan Liu

**Affiliations:** 1 Department of Life Science and Biotechnology, Nanyang Normal UniversityNanyang, Henan, 473061, China; 2 Laboratory for Conservation and Utilization of Bio-Resources, Key Laboratory for Microbial Resources of the Ministry of Education, Yunnan UniversityKunming, 650091, China; 3 Department of Pathology, School of Medicine, University of VirginiaCharlottesville, VA, 22908, USA

## Abstract

*Bacillus nematocida* B16 is able to dominate in the intestines of the worm *Caenorhabditis elegans* in ‘Trojan horse’ pathogenic mechanism. The *adp* is one candidate gene which potentially play a vital role in the colonization from our previous random mutagenesis screening results. To analyse the functional role of this gene, we constructed the *adp* knockout mutant through customized transcription activator-like effectors nucleases (TALEN), which has been successfully used in yeasts, nematodes, zebrafish and human pluripotent cells. Here, we first time report this knockout method in bacteria on this paper. Bioassay experiments demonstrated that the *adp* knockout mutant of B16 showed considerably lower colonization activity, reduced numbers of intestines and less than 80% nematocidal activity compared with the wild-type strain when infected for 48 h. However, no obvious change on proteolytic activity was observed in the mutant. Conversely, the complementation of *adp* gene restored most of the above deficient phenotypes. These results indicated that the *adp* gene was involved in surface adhesion and played a comparatively important role in colonizing host nematodes. Moreover, TALENs successfully disrupt target genes in bacteria.

## Introduction

The soil-dwelling bacteria *Bacillus nematocida* B16, a type of opportunistic pathogen, kills nematode *Caenorhabditis elegans* via a Trojan horse-like mechanism. The bacteria lured nematodes by emitting potent volatile organic compounds, entered the intestine of nematodes and caused the death of nematode by secreting two virulent proteases to destroy essential intestinal proteins (Niu *et al*., 2010). The mechanism of bacteria colonizing their host intestine during the infection process remains unclear. In our previous study, a random mutagenic B16 was used to screen mutants with impaired nematode colonization. Several potential, localization-related genes were identified (Niu *et al*., 2012). Recently, we found another novel *adp* gene, which might be associated with the colonization of bacteria B16 within their host, *C. elegans*. This *adp* gene from B16, which has a 97% similarity to the collagen-like protein, is likely involved in surface adhesion, as indicated by NCBI BLAST. Numerous data support the important role of collagen-like protein in colonization by acting as an adhesin during bacterial pathogenesis (Chen *et al*., 2010). Moreover, it has been reported that bacteria occupying intestinal adherence sites are key for successful colonization (Da Re *et al*., 2013). As a result, the *adp* gene is thought to be an important factor for colonization of strain B16. Therefore, in this study, we focus on the gene *adp* in strain B16 to investigate its role in the invasion and colonization of nematode intestines.

Recent work on highly active transcription activator-like effector nucleases (TALENs) provides an alternative approach to knock out specific genes in cells, which makes it feasible to manipulate the B16 gene in *B. nematocida*. Transcription activator-like (TAL) effectors (TALEs) are natural effector proteins secreted by numerous species of *Xanthomonas*, in order to modulate gene expression in host plants, and to facilitate bacterial colonization and survival (Boch and Bonas, 2010; Bogdanove *et al*., 2010). TALEs have revealed an elegant code linking the repetitive region of the TALEs with their target DNA-binding site (Boch *et al*., 2009; Moscou and Bogdanove, 2009). Two highly variable amino acids at positions 12 and 13, known as the repeat-variable di-residue (RVD) of mostly 33–35 amino acid tandem repeats, establish the base-recognition specificity of each unit. In detail, the corresponding nucleotide is separately NI to A, HD to C, NG to T, and NN to G or A. This strong association suggests a potentially designable protein with sequence-specific DNA-binding capabilities and the possibility of applying engineered TALEs to specify DNA binding in cells. The following are the advantages of using the TALEN method for knocking out specific genes: (i) high homologous recombination frequency, (ii) marker-free knockout of chromosome genes, (iii) genetic stability, (iv) efficient gene alteration and (v) specificity. The increasing number of studies on TALEs has prompted researchers to manipulate genomes precisely to identify DNA binding domains with high specificity in all living cells. Hockemeyer *et al*. achieved genetic manipulation in human pluripotent cells by using TALE nucleases (Hockemeyer *et al*., 2011). Tesson *et al*. successfully explored TALENs for *in vivo* genetic engineering in rats (Tesson *et al*., 2011). Miller *et al*. reported that TALENs could disrupt target genes in cultured human cells (Miller *et al*., 2011). Zhang *et al*. utilized TAL effectors to regulate endogenous gene transcription (Zhang *et al*., 2011). Huang *et al*. targeted gene modifications in zebrafish using customized TALENs (Huang *et al*., 2011). Li *et al*. applied TALENs to realize site-specific gene disruptions in native yeast chromosomal genes (Li *et al*., 2011). TALENs have also been used for gene targeting in nematodes (Wood *et al*., 2011). Even though, many papers have reported genomic modifications using TALEs, to our knowledge, none has focused on whether TALENs could be used for pro gene targeting in prokaryotic organisms. Based on the strong advantages listed above, we were prompted to utilize the TALEN technique in gram-positive bacteria. Therefore, TALENs could be used to knock out genes in *B. nematocida* B16.

Here, we designed the DNA recognition domain of TAL effectors for the distinct genomic loci of the *adp* gene, combined with the *Fok*I restriction enzyme, and produced double-strand breaks between the target sequences to disrupt the gene via non-homologous end-joining (NHEJ) and homologous recombination. The mutant strains obtained were used to test colonization capabilities compared with the wild-type strain.

## Results

### TALEN activity assay and plasmid construction

**Fig 1:**
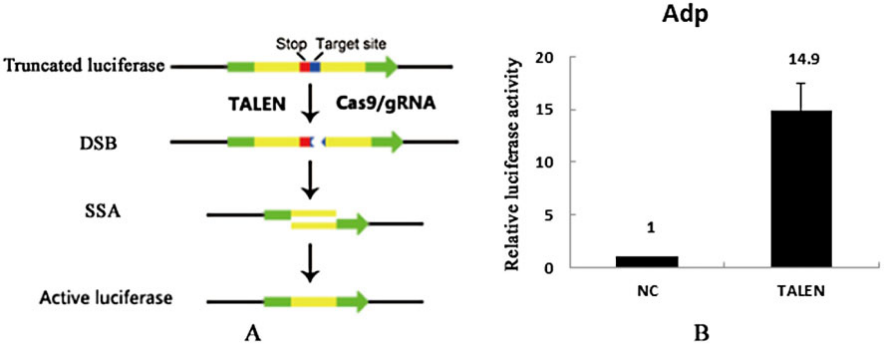
Detection of TALEN activity using luciferase SSA recombination assay.A. Diagram showing the principle of the luciferase SSA recombination assay. A stop codon (red) and a TALEN target site (blue) were placed between two truncated firefly luciferase coding regions (yellow). TALENs could induce DSB, which could in turn lead to DNA repair through SSA between the two homologous arms and result in the formation of an active luciferase. The green represents up and down stream of homologous arms of luciferase coding regions, separately.B. SSA results of TALEN targeting *adp* site. Compared with the control, the luciferase activity was increased to 14.9.

The activity of TALENs was assayed by testing the luciferase SSA recombination capabilities. The results showed that the TALENs exhibited a noticeably higher activity (14.9-fold) than the control (Fig. 1B), which indicated that constructed TALENs could be applied to the following experiments as a tool.

The plasmid pHY300-GFP originated from pHY300-Pcil, with an insterted *gfp* gene, was used by inserting a *gfp* gene to test whether the *xylA* promoter could function normally in *B. nematocida* B16. We found that *gfp* was expressed at a high level in *B. nematocida* B16 strain (data not shown), which indicated that the *xylA* promoter is constitutively active in *B. nematocida*, and leads to an efficient xylose-independent expression of heterologous open reading frames (ORFs) cloned into the vector pHY300-plk. Therefore, we chose the promoter of *xylA* to express TALEN plasmids in B16 strain. Two fragments of approximately 1.5 and 1.4 kbp were separately obtained through the digestion of the plasmids pHY300-adpL and pHY300-adpR, using two restriction enzymes, AvrII and PciI, which verified the successful construction of the recombinant plasmids.

### Identification, knockout and complementation of *adp* gene of *B. nematocida*

To identify *adp* gene encoding collagen-like protein in *B. nematocida* B16 strain, the full length of *adp* was amplified via polymerase chain reaction (PCR) and sequenced. The ORF of *adp* is 565 bp, which encodes a protein with 187 amino acid residues (data not shown). The collagen-like region of the protein Adp is composed of 38 GXX triplet repeats, which is the representative sequence of collagen protein. This observation shows that the *B. nematocida* B16 strain expresses collagen protein consisting of 38 GXX triplet repeats.

We found that the incubation period is an important factor that influences transformation frequency. Transformation efficiency was stable when the incubation period was about 60–90-min, with 90 being optimal. The efficiency of co-transformation could be up to 1300 colony-forming units (cfu) per μg DNA. Starvation probably induced the transformation efficiency of the competent cells (*B. nematocida* B16g). The bacteria were grown in a rich medium, followed by a barren medium during the proliferative stage, which caused physiological phenotype changes, including defective cell walls and cell membranes. These changes increased cell permeability and facilitated exogenous DNA entry into the cells.

In total, we obtained 52 knockout transformants. Four transformants that lost TALEN plasmids were obtained and validated using PCR and DNA Sanger sequencing. The sequences of the knockout transformants BCK16g-1, BCK16g-19, BCK16g-22 and BCK16g-43 differed from the wild-type *adp* gene (Genebank No. KC243320). The frame-shift mutation in the GACTC position is located 33 bp from the start codon, ATG. The mutant information for the four knockout transformants is shown in Table 1. The results suggest that the *adp* gene was successfully knocked out among the four transformant strains. Therefore, the knockout efficiency was calculated using the following formula: Frequency of mutagenesis = Number of knockout mutants × 100%/Total number of transformants, which is 4 × 100%/52 = 7.69%.

**Table 1:** The sequence information on the target *adp* gene among the mutant strains

No.of strains	Left target sequence (5′-3′)	Cut position	Right target sequence (5′-3′)	Mutation results
Wild type	ACTTGGTATAACAGAT	acgactcttgggattac	ACTATTCCGGCAGTC	
BCK16g-1	ACTTGGTATAACAGAT	ac-actcttgggattac	ACTATTCCGGCAGTC	(−1,fs)
BCK16g-19	ACTTGGTATAACAGAT	acgac-cttgggattac	ACTATTCCGGCAGTC	(−1,fs)
BCK16g-22	ACTTGGTATAACAGAT	acgacttctagggattac	ACTATTCCGGCAGTC	(+1,fs)
BCK16g-43	ACTTGGTATAACAGAT	ac-actcttggattac	ACTATTCCGGCAGTC	(−1,fs)

Notes: ‘−’ shows absence of base; ‘+’ indicates insertion of base; ‘fs’ expresses frame shift happening.

The plasmid pHY300-Adp was employed to rescue *adp* expression in the mutant strain BCK16g-1 through transformation. Approximately 45 complementation transformants were obtained in total. Ten out of 45 transformants were randomly selected for verification by sequencing *adp*. The *adp* sequence of the complemented mutant BCC16g-5 was exactly the same as the wild type, which is consistent with our expectation.

### Analysis on phenotype, proteolytic and bioassay activities

**Fig 2:**
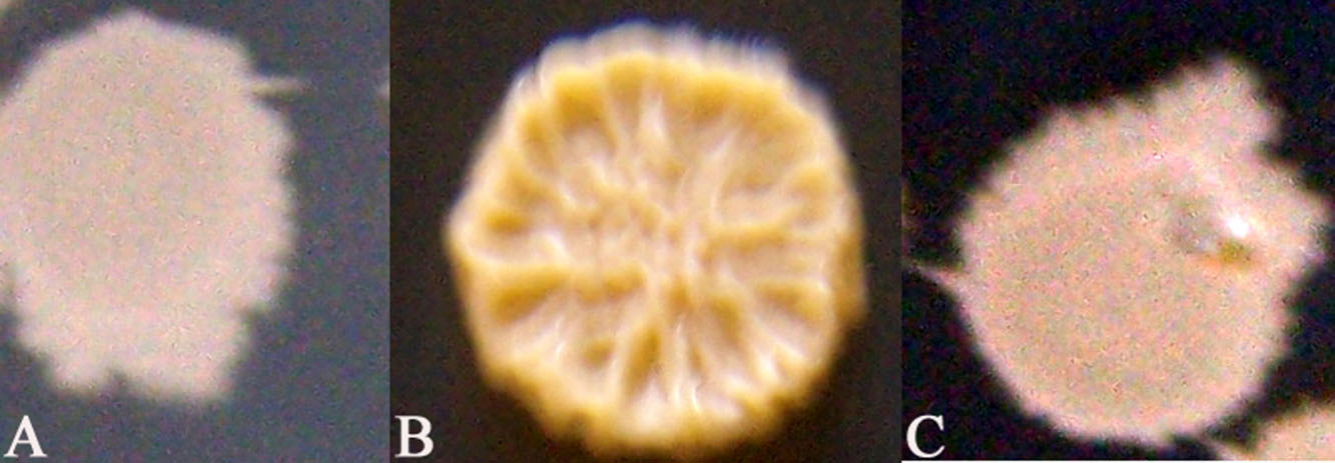
The colony morphologies of the wild-type and mutant strains on LB plates.A. Wild-type B16.B. Mutant strain BCK16g-1.C. Complement strain BCC16g-5.

When grown on Luria–Bertani (LB) plates, the *adp* gene-deleted and complemented mutants exhibited similar growth rates as the wild-type strain (data not shown). The knockout mutant strain BCK16g-1 displayed rough and raised colony surfaces. In contrast, the wild-type and complemented mutant strain BCC16g-5 displayed a smooth and flat morphology (Fig. 2). Also, compared with the wild-type and complemented mutant strains, the knockout mutant strain BCK16g-1 lost the colony thread-drawing phenomenon.

**Fig 3:**
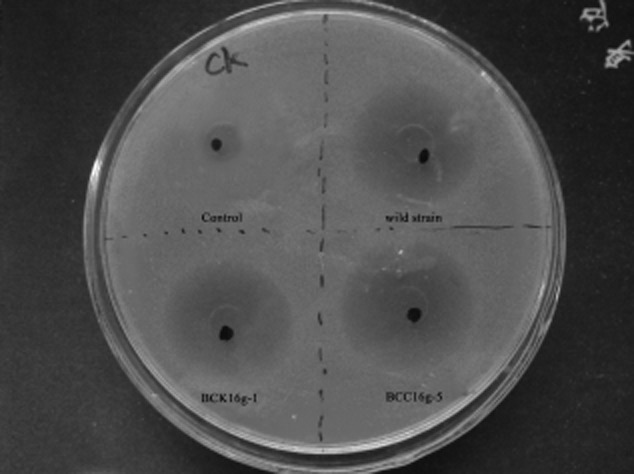
Assay of protease activity on casein plates, which produces a hydrolysis ring when degraded.

**Fig 4:**
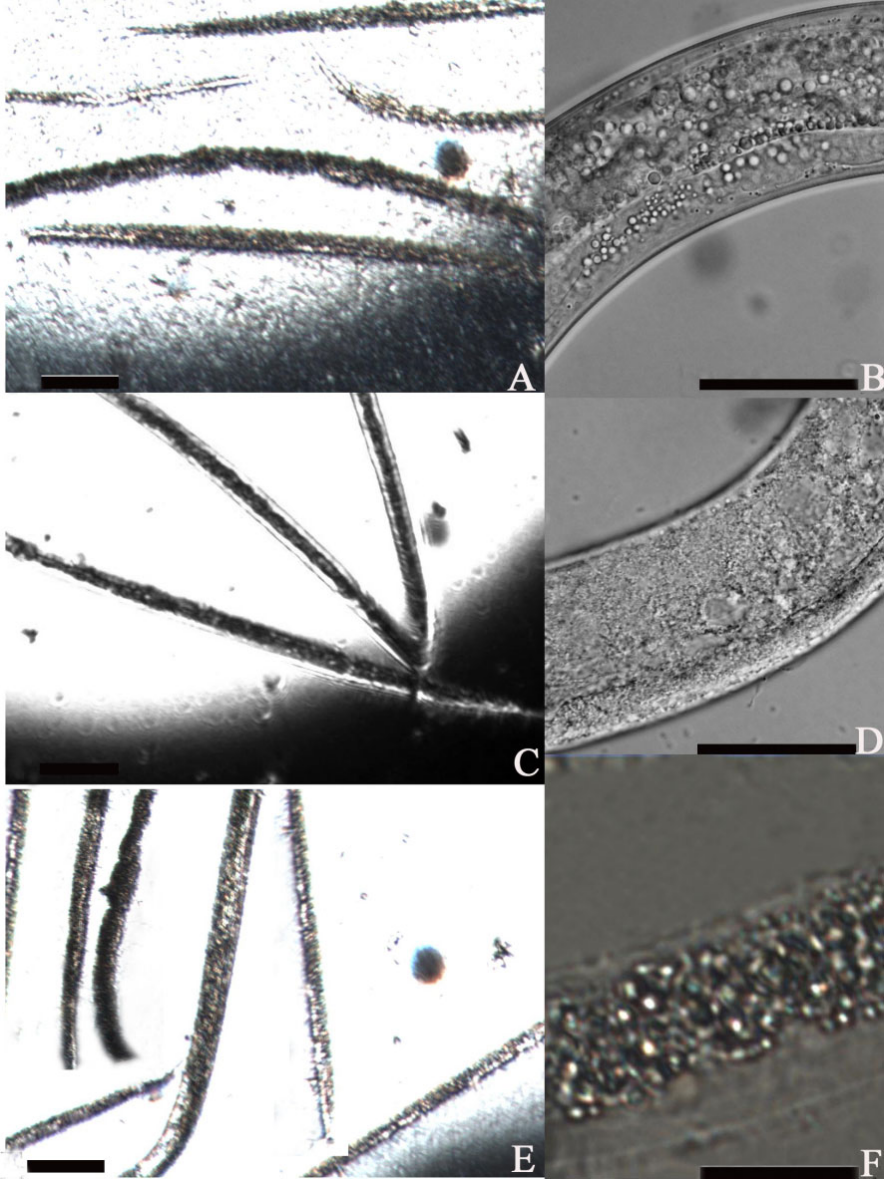
Action of the extracts from bacterial strains against nematode *C. elegans* observed under a dissecting microscope.A. Within 48 h, most nematodes were killed on the plate with *B. nematocida* B16g.B. The intestines from worms A were destroyed severely.C. The nematodes were alive on the plates incubated with BCK16g-1 after 48 h.D. The intestines from nematodes C were intact.E. Most test nematodes were dead treated with complemented strain BCC16g-5.F. The intestines from worms E were damaged badly.

To investigate the expression of the virulent proteases of the mutant strains, we first used a simplified method to estimate relative expression levels by measuring the size of the hydrolysis ring on a casein plate. We found that the mutant strain BCK16g-1 and the complemented strain BCC16g-5 produced the same size hydrolysis ring as the wild-type strain B16 when degraded. However, the two proteases (Bace16/Bae16) double-knockout strain B13, which we constructed previously (Niu *et al*., 2010), was used as a negative control and showed almost no hydrolysis circle (Fig. 3). Furthermore, the mutant strains were quantitatively examined for protease expression using a colorimetric method. The results showed that the activity of the extract source of native B16 using 0.2 M casein as a substrate was 8.2 PU × 10^−3^. The extract source of the knockout and complemented mutant strains presented a slightly lower protease activity than that of native B16g (Table 2). Our results indicate that the *adp* gene has no effect on protease activities. However, the bioassay indicated that the bacterial cells of the wild-type strain B16g and the knockout strain BCK16g-1 had remarkable differences in the ability to kill nematode *C. elegans*. The majority of the nematodes were dead after being treated with the wild-type strain B16 for 48 h, whereas only 20% of the tested nematodes were killed by the mutant strain during the same time frame. The interesting thing is that the nematicidal activity of the complemented strain BCC16g-5 was increased in comparison with the wild-type strain (Table 2). This finding suggests that the *adp* gene may play a functional role in the infection process. By using light microscope, we also observed that the intestines of the tested nematodes were completely impaired after the treatment with the wild-type strain B16g and the complemented strain BCCg16g-5 for 48 h (Fig. 4 A and E). However, the majority of the nematodes were still alive when treated with the mutant strain BCK16g-1 during this same time frame. No obvious effect could be observed on the nematode intestines (Fig. 4C and D). The intestinal tissue structure of the nematodes incubated with samples of B16g and BCCg16g-5 revealed disorganization, and lacked tight junctions (Fig. 4B and F). Compared with the defective and loose intestinal structures, the worms with BCK16g-1 had clear, complete and normal intestines. Figure 4D shows that their intestinal structure was normal and was not destroyed by the mutant strain. These results demonstrate that the killing abilities of the *adp* mutant was reduced dramatically, although it retained similar protease activities, which is consistent with the importance of colonization during pathogenesis.

**Table 2:** Comparisons of the protease and killing nematode activities between the wild type and mutant strains

Samples	Proteolytic activity of the extract source (PU) (SD)	Mortalities of nematodes (%) (SD)			
		12 h	24 h	36 h	48 h
Wild B16	8.2 (2.3) × 10^−3^	50 (1.8)	70 (3.0)	90 (2.5)	98 (4.1)
BCK16g-1	7.9 (2.0) × 10^−3^	10 (1.0)	10 (1.1)	15 (0.9)	20 (1.6)
BCC16g-5	8.0 (2.2) × 10^−3^	50 (1.7)	73 (2.1)	90 (1.4)	100 (2.9)
Water	–	3 (0)	5 (0)	5 (0.3)	7 (0.3)

### Colonization analysis

**Fig 5:**
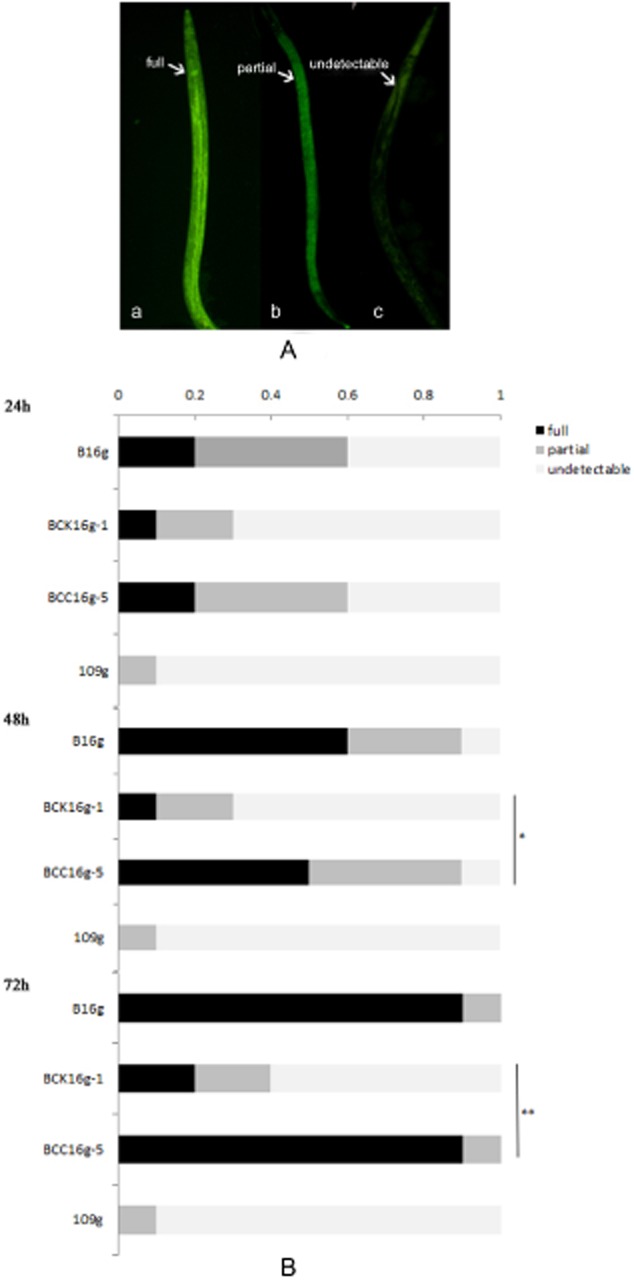
Kinetics of colonization of *C. elegans* by constant exposure to *B. nematocida*.A. Colonization categories: (a) worms evaluated as ‘full’; (b) worms evaluated as ‘partial’; (c) worms evaluated as ‘undetectable.’B. Colonization of worms by different bacterial samples. One-day-old adult wild-type worms were exposed to *B. nematocida* B16g or isogenic mutants. For each bacterial strain tested, the extent of colonization was scored in four sets of 10 nematodes every 24 h. A representative of three independent experiments with the average fraction of the population colonized for each category is shown. Chi-squared test, *P < 0.05, **P < 0.001.

Worms were visually evaluated for severity of colonization based on the extent of luminal distention and *gfp* signal in the intestine (Fig. 5A). Given that worms have a number of mechanical and chemical mechanisms for restricting bacteria in the gut, individual animals were colonized at different rates (Fig. 5B). The results showed that the mutant strain BCK16g-1 colonized *C. elegans* significantly less than those of the wild-type B16g and the complemented strain BCCg16g-5. Specifically, during the first 48 h, animals constantly exposed to the BCK16g-1 strain only had 10% scores of ‘full’ colonization. Differences between BCK16g-1 and BCCg16g-5 mutants were notable when we compared the change in the severity of colonization at 72 h (Fig. 5B, chi-squared test, *P* < 0.0001). For example, only 20% of animals that were fed with the mutant BCK16g-1 could be categorized as having ‘full’ colonization. However, 90% of animals that were fed with B16g and BCCg16g-5 were categorized in the ‘full’ colonization category. Almost no *Escherichia coli* strain 109g colonized the worm intestines throughout the entire infection process. Conversely, the wild-type strain B16g and the complemented strain BCCg16g-5 showed notably strong colonization abilities. Therefore, the *adp* gene is required to promote the *B. nematocida* colonization of the *C. elegans* intestinal lumen.

**Fig 6:**
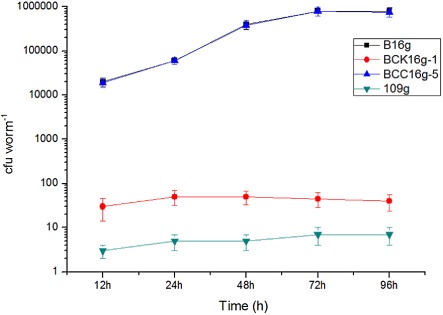
Results of cfu/worm assays by different samples.

We examined the ability of mutant strains to colonize *C. elegans* by following the kinetics of bacterial accumulation in the nematode intestine over time. The results are shown in Fig. 6. The population of intestinal *B. nematocida* reaches between 10^4^ and 10^5^ bacteria/worms in the first 3 days of infection by the strains B16g and BCCg16g-5. For worms fed with the knockout mutant strain BCK16g-1, the number of bacteria was not as high as 100 cfu/worm after being infected for 4 days, which confirmed the colonization functions of *adp* gene in intestines of *C. elegans* for pathogenic bacteria *B. nematocida*.

## Discussion

In our previous study, we found that the pathogenic bacteria *B. nematocida* B16 entered the intestines and killed nematodes by virulence extracellular alkaline serine protease, Bace16 and a neutral protease Bae16 (Niu *et al*., 2010). We proved that the colonization process occurs during pathogenesis of B16. Colonization has an important influence on killing capabilities. Bacteria that colonize the host intestines and mucous membranes share a common feature, i.e. their capability for specific adherence to surfaces. For bacteria B16, the colonization process has the characteristics of ‘social’ behaviour. How the bacteria B16 outgrew competitors and reached considerable numbers in the intestines was not well characterized. A special command system is required to modulate the processes in colonization, but few efforts work towards understanding this mechanism.

Collagen is a triple-helical, elongated protein structure that is the main structural component of the extracellular matrix in all multicellular organisms (Chen *et al*., 2010). Collagen-like sequences are found not only in proteins of multicellular organisms, but also in the proteins of microorganisms, such as a pullulanase in *Klebsiella pneuminiae* (Charalambous *et al*., 1988) and a platelet aggregation-associated protein in *Streptococcal sanguis* (Erickson and Herzberg, 1987; 1990). Collagens interact with several macromolecules and have functional significance. Many eukaryotic cells bind collagen through the integrins expressed on their surface (Camper *et al*., 1998). Lee *et al*. showed that three-dimensional collagen gel matrices combined with adhesive proteins, such as fibronectin and laminin, provided significant cues to the differentiation into neuronal lineage of mesenchymal stem cells derived from rat bone marrow (Lee *et al*., 2011). Studies have demonstrated that collagen-like peptides support human mesenchymal stem cell adhesion, spreading and proliferation (Krishna *et al*., 2011). Colace *et al*. investigated that relipidated tissue factor linked to collagen surfaces potentiated platelet adhesion and fibrin formation in a microfluidic model of vessel injury (Colace *et al*., 2011). Lenting *et al*. reported that collagen is a main initiator for platelet adhesion and aggregation, and that collagen binds to leucocyte-associated Ig-like receptor-2 efficiently inhibiting platelet activation and adhesion (Lenting *et al*., 2010). *Streptococcus pyogenes* causes heterogeneous disease types, in addition, this bacterium has been reported to produce a number of surface-associated and extracellular products that contribute to pathogenesis (Stevens, 1992; Norrby-Teglund and Kotb, 2000). Among these products, collagen-like surface protein on *S. pyogenes* was determined to promote adhesion to respiratory epithelial cells, and was documented as being involved in adherence and colonization during infection (Hasty *et al*., 1992; Chen *et al*., 2010). The research above shows that collagen proteins play important roles in cell adhesion.

Cell adhesion to the extracellular matrix is mediated by focal adhesions, which are specialized structures involved in the coupling of cytoskeletal elements to membrane receptors and in the recruitment of signalling complexes (Berrier and Yamada, 2007). The assembly of focal adhesions is considered a relevant test for analyzing *in vitro* biocompatibility (Owen *et al*., 2005). We attempted to identify such structures of collagen-like protein ADP in B16 cells by bioinformatics. No similar structure could be found in 3D macromolecular structure databases. The protein sequence of ADP has 82% similarity with a hypothetical protein in *Bacillus amyloliquefaciens* and 67% identity with collagen-like protein in *Bacillus thuringiensis*. The function of these proteins remains unreported.

The average mutation rate involving the TALEN-mediated inactivation method of a target gene is 25% according to our experience in animal cell gene knockout. The mutation occurred during the DNA NHEJ repair process, after cutting the aim gene fragment by the endonuclease. The TALE construct is designed near the endonuclease. If a mutant is formed, the enzyme sites are destroyed and cannot be cut. Therefore, the mutant strain was investigated by digesting the PCR product. Finally, the mutants were confirmed by sequencing the PCR products. During our experiments, we directly used DNA sequencing methods to confirm the occurence of mutants due to the lower number of transformants. TALEN-mediated mutants mainly have frame-shift mutations. The protein products of these genes do not exist due to frame-shift mutations. Thus, the target position of TALE should be close to the start codon, ATG, to ensure that the target gene was completely knocked out via the NHEJ pathway. This is the best method used to apply TALENs in bacteria. Otherwise, the transfomant cell cannot survive, as the DNA double-strand breaks caused by TALE are lethal.

In this study, we confirmed the function of the *adp* gene, which is related to colonization of *C. elegans* by *B. nematocida* B16. Also, we successfully reported a new and efficient technique to obtain mutants of the *adp* gene in *B. nematocida* B16 through TALENs, for the first time, in prokaryotic cells. According to our experimental results, the mutation frequency could be as high as 7.69%. Compared with the knockout efficiency in animal cells (average 25%) using TALEN technology, we obtained a comparatively lower mutation rate. The reason is probably due to the low transformation efficiency in *Bacillus*. Even so, our research may provide a strategy for others who may wish to apply TALENs in other bacteria. Our results underscore the importance of *adp* in the colonization of *B. nematocida* B16 in the intestinal epithelial cells of the worm *C. elegans*. Also, our research gives new data to clarify the mechanisms used by *B. nematocida* to colonize the intestines of the nematodes as a parasitic microorganism. Understanding the mechanisms by which *B. nematocida* colonize the intestinal cells may lead to alternative therapeutic methods for decolonization and decrease the dependence on antibiotics.

## Experimental procedures

### Strains, plasmids and growth conditions

The bacterial strains and plasmids used in this study are listed in Table 3. The strains were cultured as follows.

**Table 3:** Bacterial strains and plasmids

Strains/Plasmids	Description	Reference/Source
Strains		
*Bacillus nematocida* B16	Original pathogenic bacteria	CGMCCC (catalogue 1128)
*B.nematocida* B16g	GFP-expressing strain	Niu *et al*. (2012)
*B.nematocida* BCK16g-1	B16g derivative with deletion in *adp* gene	This study
*B.nematocida* BCC16g-5	Complementation of mutant strain BCK16g-1	This study
*Escherichia coli* JM109	recA1,endA1,gyrA96,thi-1,hsdR17,supE44, relA1,.Δ lac-proAB)/F′[traD36, proAB, lacIq, lacZ.M15]	Ausubel *et al*. (1994)
*E.coli* 109g	JM109 derivative expressing GFP	Niu *et al*. (2010)
Plasmids		
pMD18-T	Col E1 origin, T-vector; Amp^r^	Takara Co.
pHY300-plk	ori-pAM α1,ori-177,Amp^r^,Tet^r^	Dr. Qinggang Guo of Hebei Academy of Agricultural and Forestry Sciences
pAX01	ori,lacA′-′lacA,xylR,P_xylA_,Amp^r^,Ery^r^	Bacillus Genetic Stock Center
pHY300-Pcil	Derived from pHY300-plk,containing *xylA* promoter of pAX01	This study
pHY300-GFP	Derived from pHY300-Pcil with *gfp* located on the downstream of *xylA* promoter	This study
pHY300-Adp	Derived from pHY300-Pcil with *adp* located on the downstream of *xylA* promoter	This study
pCS2-peas-T	Expression vector in animal cells containing TALEN-Adp-L	Viewsolid Biotech Co. Ltd
pCS2-peas-R	Expression vector in animal cells containing TALEN-Adp-R	Viewsolid Biotech Co. Ltd
pHY300-adpL	pHY300-Pcil containing TALEN-Adp-L;Amp^r^,Tet^r^	This study
pHY300-adpR	pHY300-Pcil containing TALEN-Adp-R;Amp^r^,Tet^r^	This study

GFP-expressing strain B16g was grown at 37°C in LB medium with 5 μg ml^–1^ chloramphenicol, and was used as the parent strain for the derivation of the *adp* knockout mutant. The strain was grown at 30°C in YPD broth containing 1% yeast extract, 2% peptone and 2% glucose 25 with shaking (200 r min^−1^) for 3 days for production of proteases. Unless otherwise indicated, *E. coli* was grown at 37°C on LB agar, or in LB broth with shaking, supplemented with the appropriate antibiotic. *E. coli* JM109 was used as a host organism to carry pMD18-T vector or its derivatives.

### Culture of nematodes

The tested nematodes *C. elegans* were grown on standard growth medium (NGM: 0.25% peptone, 51 mM NaCl, 25 mM K_3_PO_4_, 5 μg ml^–1^ cholesterol, 1 mM CaCl_2_ and 1 mM MgCl_2_) plates seeded with *E. coli* strain 109g as the food source at 25°C for 24 h (Brenner, 1974). The nematodes were synchronized to the L4 stage while performing all experiments. Nematodes were washed thoroughly with sterile water before being utilized in the assays.

### TALEN constructs

**Fig 7:**
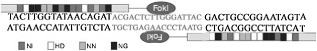
Diagram of design of the TALENs that target the *adp* locus.

The *adp* gene sequence of B16 was submitted to NCBI (Accession number: KC243320). The DNA recognition domain can be combined with the nuclease domain of the *Fok*I restriction enzyme to produce TALENs. Figure 7 shows the design of TALENs targeting the *adp* locus. The specific repeat-variable di-residue used to recognize each base is indicated by shading, as defined in the key. A thymidine nucleotide (T) is present at the 5′ end of each binding site. The TALEN plasmids were purchased from Viewsolid Biotech (Beijing, China). The activity of the TALEN plasmids was investigated by using a luciferase single strand annealing (SSA) recombination repair detection kit (Catalog. No. VK002, Viewsolid Biotech) as described by Bhakta and Segal (2010) (Fig. 1A). The relative luciferase activity was detected using a dual-luciferase assay system (Promega) and measured using a SpectraMax luminescene microplate reader (Molecular Device) to test TALEN activity.

### Plasmid reconstruction

**Fig 8:**
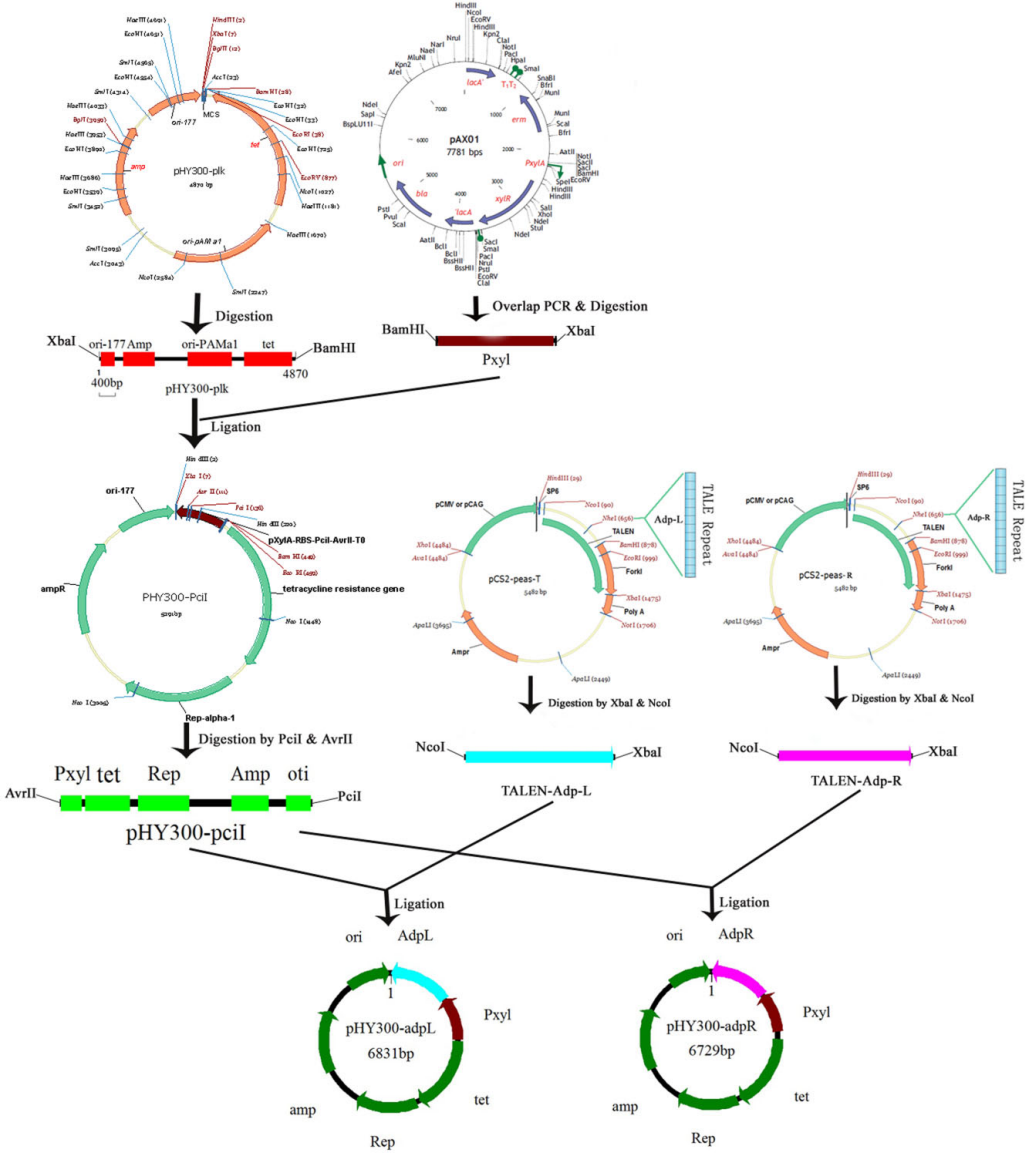
Diagram of the detailed process of a pair of TALEN plasmid reconstruction.

The regulatory elements of the xylose (xyl) operon are commonly used to control the production of recombinant genes in *Bacillus*. First, overlapping PCR was carried out to obtain the fragments of the promoter of *xylA*, which is a ribosome binding site (RBS), and the termination sequence T0. Two pairs of oligonucleotide primers, namely, Pxyl-FP: TATATATGGATCCCATTTCCCCCTTTG, which contains the BamHI restriction site (underlined); Pxyl-pciI-RP:AAAAGCTGAAAGATCTTAACATGTTTGACACCTCCTTTCGATCTGCAATTTGAATAATA, which contains the PciI restriction site (underlined); To-pciI-FP: GTGTCAAACATGTTAAGATCTTTCAGCTTTTACCTAGGGAGCTCCCCGGGACGTTCTTG, which contains the AvrII restriction site (underlined); and To-pciI-RP: TATATATTCTAGATCGATATCTCTGCAGTCGCG, which contains the XbaI restriction site (underlined), were designed based on the nucleotide sequence of PxylA in plasmid pAX01. The detailed process of plasmid reconstruction is shown in Fig. 8. The overlapping PCR-amplified 448 bp DNA fragment was digested using BamHI and XbaI, and then ligated into the vector pHY300-PLK digested using the same restriction enzymes to generate plasmid pHY300-PciI. To confirm the activity of the *xylA* promoter in *B. nematocida*, we inserted the *gfp* gene downstream of the promoter *xlyA* in the plasmid pHY300-Pcil to test *gfp* expression level. The fragments of TALEN-Adp-L and TALEN-Adp-R were obtained by digesting the plasmids, pCS2-peas-T and pCS2-peas-R, by using the restriction endonucleases NcoI and XbaI. The two fragments were individually inserted into the AvrII and PciI restriction enzyme sites of the plasmid pHY300-PciI. The constructed plasmids were named pHY300-adpL and pHY300-adpR respectively. The plasmids contained the promoter of *xylA*, RBS, TALEN and the termination sequence T0. The two plasmids pHY300-adpL and pHY300-adpR were used for subsequent transformation experiments.

### Targeted gene disruption of *adp* in bacteria

The plasmids pHY300-adpL and pHY300-adpR were co-transformed into the competent cells of *B. nematocida* B16 by competence transformation according to the protocols supplied by BGSC (*Bacillus* Genetic Stock Center, department of Biochemistry, The Ohio State University, Columbus, Ohio, USA) (Anagnostopoulos and Spizizen, 1961). Transformant clones were screened for plasmid-associated properties by the LB medium containing 25 μg ml^−1^ of tetracyclines. The transformants were incubated on LB medium with tetracyclines for three continuous transfers to screen the stable clones. Primers adkfor (5-ATGAGAAAAGGAGATACTTGG-3) and adkrev (5-TTATCCCAGTGGCTCCGG-3) were designed based on the known collagen-like protein sequence (KC243320), and used to amplify the *adp* gene in transformants. The PCR productions were sequenced to validate the knockout of the gene in the genome. The frame-shift mutation transformants confirmed by sequencing were incubated on the LB plate without tetracyclines for at least 15 continuous transfers to discard the TALEN plasmids. The objective clones that lost tetracycline-resistance were our final mutants.

### Complementation of the *adp* deletion mutant

The *adp* gene was amplified by PCR with an NcoI-linked sense primer, 5′-CATGCCATGGATGAGAAAAGGAGATACTTG-3′, and an XbaI-linked antisense primer, 5′- TGCCTCTAGATCCCAGTGGCTCCGGTTAC-3′ from the genomic DNA of the wild strain. The amplified product was purified, double digested with NcoI and XbaI, and inserted into the expression plasmid pHY300-PciI, resulting in pHY300-Adp. For complementation studies, the mutant strain was transformed with plasmid pHY300-Adp, which carried an ORF of *adp* gene under the control of the *xylA* promoter. The positive mutant colonies were selected on LB plates containing 20 μg ml^−1^ tetracycline. The identity of the clones were confirmed by PCR and sequencing.

### Measuring the proteolytic, nematotoxic and colonization activities

The colonies of bacterial strain were inoculated into yeast extract/peptone/dextrose (YPD) medium in flasks and cultured at 37°C for 3 days under shaking (200 r.p.m.). The culture filtrates were centrifuged at 8000 r.p.m. for 10 min at 4°C, and the supernatant collected was subjected to 85% ammonium sulfate saturation by slow continuous stirring at 4°C. The solution was left overnight at 4°C, followed by centrifugation at 8500 r.p.m. for 30 min at 4°C. Subsequently, the precipitate was dissolved in a minimum amount of 50 mM sodium phosphate buffer (pH 7.5). Following dialyses, this resultant sample was designated as the crude protease extract and was tested for proteolytic activities according to the literature (Niu *et al*., 2006).

The nematotoxic activities by bacteria were performed according to the modified dialysis membrane technique (Rosen *et al*., 1997). In brief, cellophane paper was used to cover YPD medium plates to keep nematodes from moving into the medium. Bacteria were inoculated onto the cellophane paper and incubated at 28°C for 7 days to 10 days. A total of 150 tested nematodes were placed in the middle of the plate. Each plate was plotted into 20 panes, and the mortality of nematodes was counted in 5 of 20 panes stochastically every 12 h. The nematodes were considered dead when no movement was observed under a light-dissecting microscope, and when gentle tapping of nematodes by a stick did not result in movement. The experiments were performed with three parallels and in triplicate.

The kinetics of colonization of *C. elegans* through constant exposure to bacteria was determined under a Nikon 800 Eclipse microscope (Nikon Corporation, Japan) equipped for epifluorescence with a mercury lamp and an excitation filter of 450–490 nm (blue light) and a barrier filter of 515 nm at 200× magnification using the method described by Alegado and Tan (2008). At each time point, three sets of 10 infected worms were selected to evaluate the colonization situations. The worms with fluorescent bacteria in the entire lumens were evaluated as full; worms without any green fluorescence signal in the lumen were evaluated as undetectable; and worms harbouring bacteria between these two extremes were evaluated as partial.

The number of colonizing bacteria within the *C. elegans* digestive tract was measured using the method described in previous literature (Garsin *et al*., 2001; Alegado and Tan, 2008). Three sets of 10 infected worms were picked at each time point, tested and surface sterilized by placing them on an agar plate that contains 100 μg ml^–1^ of gentamicin. The worms were washed using an M9 buffer that contains 100 μg ml^–1^ of gentamicin and 25 mM of levamisole to paralyze and inhibit pharyngeal pumping and expulsion and to prevent gentamicin from entering the intestinal lumen. This procedure also releases luminal bacteria. The animals were washed twice more using an M9 buffer alone and homogenized using M9 that contains 1% Triton X-100 to recover bacteria within the worm intestine. Subsequently, the washed nematodes were mechanically disrupted using a grinding rod. After appropriate diluting, the lysates were plated onto selective media. Each data point represents the mean cfu from triplicate samples, and the error bars represent the standard deviation.

All percentage values represent w/v in the experimental procedures.

## Conflict of interest

None declared.
